# Immunization with Recombinant TcdB-Encapsulated Nanocomplex Induces Protection against *Clostridium difficile* Challenge in a Mouse Model

**DOI:** 10.3389/fmicb.2017.01411

**Published:** 2017-07-25

**Authors:** Yi-Wen Liu, Yu-Hung Chen, Jenn-Wei Chen, Pei-Jane Tsai, I-Hsiu Huang

**Affiliations:** ^1^Department of Microbiology and Immunology, College of Medicine, National Cheng Kung University Tainan, Taiwan; ^2^Department of Biochemistry and Molecular Biology, College of Medicine, National Cheng Kung University Tainan, Taiwan; ^3^Center of Infectious Disease and Signaling Research, National Cheng Kung University Tainan, Taiwan; ^4^Department of Medical Laboratory Science and Biotechnology, College of Medicine, National Cheng Kung University Tainan, Taiwan

**Keywords:** *Clostridium difficile*, TcdB, nanocomplex adjuvant, vaccine

## Abstract

*Clostridium difficile* is considered to be one of the major cause of infectious diarrhea in healthcare systems worldwide. Symptoms of *C. difficile* infection are caused largely by the production of two cytotoxins: toxin A (TcdA) and toxin B (TcdB). Vaccine development is considered desirable as it would decrease the mounting medical costs and mortality associated with *C. difficile* infections. Biodegradable nanoparticles composed of poly-γ-glutamic acid (γ-PGA) and chitosan have proven to be a safe and effective antigen delivery system for many viral vaccines. However, few studies have used this efficient antigen carrier for bacterial vaccine development. In this study, we eliminated the toxin activity domain of toxin B by constructing a recombinant protein rTcdB consists of residues 1852-2363 of TcdB receptor binding domain. The rTcdB was encapsulated in nanoparticles composed of γ-PGA and chitosan. Three rounds of intraperitoneal vaccination led to high anti-TcdB antibody responses and afforded mice full protection mice from lethal dose of *C. difficile* spore challenge. Protection was associated with high levels of toxin-neutralizing antibodies, and the rTcdB-encapsulated NPs elicited a longer-lasting antibody titers than antigen with the conventional adjuvant, aluminum hydroxide. Significant reductions in the level of proinflammatory cytokines and chemokines were observed in vaccinated mouse. These results suggested that polymeric nanocomplex-based vaccine design can be useful in developing vaccine against *C. difficile* infections.

## Introduction

*Clostridium difficile* is a Gram-positive, anaerobic spore-forming bacterium and is the leading cause of antibiotic-associated diarrhea within hospital settings worldwide (Ananthakrishnan, 2011). It has been estimated that *C. difficile* infections (CDI) are responsible for 15–25% of all antibiotic-associated diarrhea (Bartlett, 2008). Disruptions of the hosts’ microbiota by broad-spectrum antibiotic treatments, such as clindamycin, or alteration in the endogenous gastrointestinal flora are considered major risk factors for *C. difficile* infection (Bartlett, 2008; Ananthakrishnan, 2011). CDI can result in a wide spectrum of signs ranging from asymptomatic colonization, mild to severe chronic diarrhea, pseudomembranous colitis, and even death due to multiple organ failures (Dobson et al., 2003; Aslam and Musher, 2006). Treatment of CDI mainly relies on the use of metronidazole and vancomycin, although increasing cases of treatment failure or multiple relapses have raised concern over the need for alternative treatments (Ananthakrishnan, 2011). Furthermore, since treatment still relies on antibiotic usage, the normal flora is not easily restored. In addition, *C. difficile* spores can be present in the hospital setting, thus multiple relapses are quite common and making effective treatment difficult (Johnson, 2009). In recent years alternative therapeutic approaches such as fecal material transplantation (FMT) have gained ground as being effective and patients experience fewer relapses due to the recolonization of the intestinal microbiota (Borgia et al., 2015). However, safety issues can still exist with FMT due to the lack of knowledge of the effective component within the fecal sample (Borgia et al., 2015). Therefore, a vaccine approach is highly desired.

*Clostridium difficile* infections is a toxin-mediated intestinal disease. Biochemical and molecular studies have shown that the major virulence factors of toxigenic *C. difficile* are the large secreted glucosyltransferase protein toxins TcdA and TcdB, which are encoded within the PaLoc locus (Braun et al., 1996; Awad et al., 2014). Collectively the toxins act on the intestinal epithelium of the host and stimulate intestinal fluid secretion and proinflammatory responses that lead to diarrhea and colitis. The respective roles of TcdA and TcdB have been extensively studied. Carter et al. (2012) demonstrated that TcdB is the major virulence factor and TcdB alone was sufficient to induce severe organ damages *in vivo* (Carter et al., 2015). However, other studies using mutants have shown that strains expressing only TcdA retained virulence (Kuehne et al., 2010). Clinically, while naturally occurring TcdA – TcdB + strains have been isolated frequently from patients, few cases have been reported of naturally occurring TcdA + TcdB – strains in literature (Johnson et al., 2003; Monot et al., 2015). Nevertheless, both TcdA and TcdB are immunogenic and have been used as candidate antigens for the majority of vaccine studies to date (Zhao S. et al., 2014; Kociolek and Gerding, 2016).

Both TcdA and TcdB share similar C-terminal receptor binding domains (RBDs) that mediate the binding of toxins to carbohydrate receptors on the surface of host cells (Di Bella et al., 2016). Past immunization studies using the RBDs of *C. difficile* toxins have been shown to induce antibody responses with toxin-neutralizing activity in mice or hamsters challenged with either toxins or live bacteria (Baliban et al., 2014; Maynard-Smith et al., 2014; Guo et al., 2015; Huang et al., 2015; Wang et al., 2015; Bezay et al., 2016).

A critical component of any vaccine is the adjuvant. Adjuvants are essential for enhancing and directing the adaptive immune response to vaccine antigens (Leroux-Roels, 2010). The most common and traditional adjuvant for human vaccines is aluminum salt (Alum) which has been in use for about 90 years (Glenny, 1930). Other non-mineral salt based adjuvants such as lipid particles, microparticles, immune potentiators and natural polymers have also been extensively tested in pre-clinical or clinical trials (Reddy et al., 2007; Leroux-Roels, 2010; Karch and Burkhard, 2016; Kalam et al., 2017). Among these adjuvants, natural polymer based nanoparticles, which has been used ad drug delivery systems, have also shown to be a safe and effective vaccine adjuvant (Mishra et al., 2010; Moon et al., 2012; Zhao K. et al., 2014). However, the effectiveness of using nanoparticles as *C. difficile* vaccine adjuvant have not been studied in the past. In this study, we evaluated a nanoparticle vaccine consisted of recombinant TcdB RBD encapsulated by a mixture of chitosan and poly-γ-glutamic acid (γ-PGS) for the ability to induce neutralizing antibodies and to protect mice from lethal *C. difficile* spore challenge.

## Materials and Methods

### *Clostridium difficile* and *Escherichia coli* Culturing Conditions

*Clostridium difficile* strains were cultured anaerobically on brain-heart infusion (BHI) agar or in BHI broth (Thermo Scientific, Waltham, MA, United States) supplemented with 0.05% L-cysteine. Anaerobic experiments were conducted inside a Don Whitley DG250 anaerobic workstation (Don Whitley Scientific Ltd, West Yorkshire, United Kingdom). *E. coli* strains were grown at 37°C in LB (Luria Broth, Thermo Scientific, Waltham, MA, United States).

### Spore Preparations

Spores were prepared by plating a 1:100 dilution of overnight culture onto BHIS agar plates and then incubated for 10 days at 37°C under anaerobic conditions. Spores were harvested with ice-cold sterile distilled water and purified with 50% Nicodenz (Axis Shield, Oslo, Norway) as previously described (Sorg and Sonenshein, 2008). Spores were purified to >99% purity as determined by phase contrast microscopy and the number of spores/ml was quantified by visual enumeration using a Neubauer Chamber (Sigma–Aldrich, St. Louis, MO, United States). Spores were stored at -80°C and viability were confirmed by plating onto BHI agar containing the germinant sodium taurocholate prior to use.

### Protein Overexpression and Purification

Recombinant TcdB antigen was constructed based on part of the TcdB RBD (amino acids 1852-2363). Genomic DNA of *C. difficile* strain R20291 was used in a PCR reaction with primers tcdB-F and tcdB-R (**Table 1**). Restriction endonuclease sites (EcoRI at the 3′-end and HindIII at the 5′-end) were designed into the primers. The PCR-amplified product was subsequently cloned into the pET21B expression vector (Merck Millipore, Darmstadt, Germany) and transformed into *E. coli* BL21 (DE3). The sequence of the recombinant plasmid was confirmed by a commercial sequencing company (Genomics, Taiwan). Expression of recombinant 6xHis-tagged TcdB protein was induced by adding 0.5 mM isopropyl-β-D-thiogalactopyranoside (IPTG) when the cells reached an O.D._600_ of 0.6, and further incubated at 37°C for 4 h. Cells were centrifuged at 8000 rpm for 30 min at 4°C, resuspended in phosphate buffer saline (PBS; 137 mM NaCl, 2.7 mM KCl, 10 mM Na2HPO4, 2 mM KH2PO4, pH 6.5), and disrupted by sonication on ice. Next, the supernatants were loaded into an Ni-NTA column (GE Healthcare Life Sciences, Pittsburg, PA, United States) and contaminant proteins were eliminated through a washing procedure by using 50 mM imidazole in wash buffer (20 mM NaH2PO4, 500 mM NaCl, 50 mM imidazole, pH 6.5). Proteins were eluted with 500 mM imidazole in wash buffer. After SDS-PAGE analysis, proteins were concentrated by Amicon^®^ Ultra 30-kDa cut-off unit (Merck Darmstadt, Germany). The 6xHis-tag of rTcdB was removed by the Thrombin CleanCleave^TM^ kit (GE Healthcare Life Sciences, Pittsburg, PA, United States) according to the manufacturer’s instructions. Tag-less recombinant proteins were verified by Western blots. All recombinant proteins were subsequently stored at 4°C for future use.

**Table 1:** Sequences of oligonucleotide primers used in this study.

Name	Sequence (5′ to 3′)	Species	Reference
mIL-1β-F	GCA ACT GTT CCT GAA CTC AAC T	Mouse	Hung et al., 2015
mIL-1β-R	ATC TTT TGG GGT CCG TCA AT	Mouse	Hung et al., 2015
mIL-17A-F	GCT CCA GAA GGC CCT CAG A	Mouse	Hung et al., 2015
mIL-17A-R	CTT TCC CTC CGC ATT GAC A	Mouse	Hung et al., 2015
mIL-6-F	AGG ATA CCA CTC CCA ACA GAC	Mouse	Hung et al., 2015
mIL-6-R	GTG CAT CAT CGT TGT TCA TAC	Mouse	Hung et al., 2015
mTNFα-F	CAT CTT CTC AAA ATT CGA GTG ACA A	Mouse	Hung et al., 2015
mTNFα-R	TGG GAG TAG ACA AGG TAC AAC CC	Mouse	Hung et al., 2015
mMIP-2-F	TGT CAA TGC CTG AAG ACC CTG CC	Mouse	Hung et al., 2015
mMIP-2-R	AAC TTT TTG ACC GCC CTT GAG AGT GG	Mouse	Hung et al., 2015
mIFN-r-F	GCC ATC AGC AAC AAC ATA AGC GTC	Mouse	Hung et al., 2015
mIFN-r-R	CCA CTC GGA TGA GCT CAT TGA ATG	Mouse	Hung et al., 2015
mMCP-1-F	CCC ACT CAC CTG CTG CTA CT	Mouse	Hung et al., 2015
0	TCT GGA CCC ATT CCT TCT TG	Mouse	Hung et al., 2015
slpA (EcoRI)-F	TAC GAATTCG GCA GAA GAT ATG TCG AAA GTT GAG	*C. difficile*	This work
slpA (HindIII)-R	ACC AAGCTT ACT CTT AGT TGT AAC TCT TTT TCC	*C. difficile*	This work
tcdB (EcoRI)-F	TAC GAATTCG TTG ATA ACT GGA TTT ACA ACT	*C. difficile*	This work
tcdB (HindIII)-R	ACC AAGCTT CAC TAA TTG AGC TGT ATC AGG	*C. difficile*	This work

### Preparation and Characterization of Empty and Antigen Containing Nanoparticles

The antigen loaded chitosan/γ-PGA NPs were prepared by flush mixing of an aqueous γ-PGA (1 ml, unfractionated γ-PGA) into an aqueous chitosan (pH 6.0, 5 ml) at various weight ratios under magnetic stirring at room temperature (Lin and Chen, 2017). The obtained nanoparticle solution was then dialyzed (MWCO: 10000, Spectrum Labs, Rancho Dominguez, CA, United States) against deionized water for 3 days. The stock solutions of chitosan and γ–PGA were prepared by mixing γ-PGA with chitosan solution. After vacuum drying, nanoparticles were homogenized with phosphate-buffered saline (PBS, pH 7.0). The particle size, polydispersity index (PDI) and zeta potential of the prepared nanoparticles were measured using a Zetasizer (3000HS, Malvern Instruments, Malvern, United Kingdom). For the preparation and characterization of polymer-based nanoparticle encapsulated rTcdB, rTcdB protein was premixed with aqueous γ-PGA and added into aqueous chitosan under magnetic stirring in 10 mM phosphate buffer (pH 6.0) at room temperature. The samples were concentrated to 1/10 of volume and stored at 4°C. The particle size and zeta potential of the prepared nanoparticles were measured using a quasi-elastic light scattering (QELS) analyzer (3000HS, Malvern Instruments, Malvern, United Kingdom).

### Mice Immunization and Sample Collection

Specific-pathogen-free 6-weeks old male C57BL/6 mice were housed in the Laboratory Animal Center of National Cheng Kung University. All mice were maintained and handled according to the guidelines of the Institutional Animal Care and Use Committee (IACUC) of National Cheng Kung University (NCKU). All animal studies were performed following a protocol approved by the IACUC of NCKU (approval NCKU-IACUC-102-149). Mice were vaccinated intraperitoneally every other week for a total of three injections. For optimization of nanoparticle sizes, eight groups of three mice each were injected intraperitoneally with the following inoculant: (1) sterile PBS control; (2) NPs only (200 nm); (3) purified rTcdB; (4) NP_200_; (5) NP_350_; (6) NP_500_; (7) NP_750_; (8) 100 μg of rTcdB in PBS mixed with aluminum hydroxide [Al(OH)3; 1:1 by volume] (Thermo). For *C. difficile* challenge experiments, mice in groups of 5 were vaccinated with the following: (1) sterile PBS control; (2) empty NPs only; and (3) NP_750._ A total of 20 μg of rTcdB were administered to each mouse per injection. All injections were performed intraperitoneally. Serum samples were collected from each animal via submandibular collection 1 week after each vaccination and stored at -80°C prior to use.

### Detection of Vaccine-Induced Specific IgG and IgA by ELISA

TcdB-specific IgA and IgA were determined by enzyme-linked immunosorbent assay (ELISA). Purified rTcdB proteins were coated onto ELISA plates (Nunc, Roskilde, Denmark) using coating buffer (20 mM NaCO3, 35 mM NaHCO3, pH 9.6) at 4°C overnight. The wells were then blocked with 10% skim milk in PBS (pH 7.4) at room temperature. To detect the antigen-specific antibody, mouse serum samples were diluted in PBS and incubated for 1 h at 37°C. Plates were washed 3 times with 0.05% Tween 20 in PBS (PBS/T) and then incubated with HRP-conjugated anti-mouse IgG or IgA for 1 h at 37°C. Colors were developed by tetramethylbenzidine substrate (TMB) and the reaction stopped by adding stop solution (2M H_2_SO_4_). Absorbance was measured at 450 nm using iMark^TM^ Microplate Reader (Bio-Rad, Hercules, CA, United States).

### Cell Culture

Vero cells were cultured in Dulbecco’s modified Eagles medium (DMEM) containing Penicillin-Streptomycin and 10% fetal bovine serum (FBS) in a humidified incubator containing 5% CO_2_ at 37°C. Cells were detached using 1000 U/ml trypsin and 0.5 mM EDTA and counted by LUNA-FL^TM^ Dual Fluorescence Cell Counter (Logos Biosystems, Anyang, South Korea). Then cells were seeded into a 96 well tissue culture test plate (SPL life sciences, Pocheon, South Korea) at a density of 5 × 10^4^ cells per well and incubated at 37°C containing 5% CO_2_ overnight.

### Neutralizing Antibody Assay

Toxin neutralizing properties of antiserum were determined using Vero cells and *C. difficile* purified toxin B (List Biological Labs, Campbell, CA, United States). Serum samples obtained from all immunized mice were serially diluted in DMEM and incubated for 1 h at 37°C with toxin B (final concentration 0.5 ng/ml). The toxin-serum mixtures were then added to 96 well plates containing Vero cells and the plates were incubated at 37°C containing 5% CO2 for 18 h. After incubation, the culture supernatant was collected and incubated with the substrate mixture from the Cytotoxicity detection kit (Roche, Basel, Switzerland) for 30 min in dark. The lactate dehydrogenase (LDH) activity was then determined at 492 nm using iMark^TM^ Microplate Reader (Bio-Rad, Hercules, CA, United States), and cytotoxicity was calculated from the following equation. Statistical analyses were performed using GraphPad Prism version 6.0.

$$\begin{array}{c} \mathrm{Cytotoxicity}\;(\%)\;=\frac{\left(\exp.value-background)-(\mathrm{low}control-background\right)}{\left(\mathrm{high}control-background)-(\mathrm{low}control-background\right)}\;\times \;100\% \end{array}$$

Background control = medium only + reagent only

Low control = spontaneous LDH release

High control = maximum LDH release

### Animal Model of CDI

After pre-vaccination, mice were fed drinking water containing an antibiotic mixture, which included 0.4 mg/ml vancomycin, 0.215 mg/ml metronidazole, 0.4 mg/ml kanamycin, 0.035 mg/mL gentamycin, and 850 U/ml colistin, for a total of 5 days before challenge. All antibiotics were purchased from Sigma-Aldrich. Vancomycin and metronidazole were omitted to avoid disrupting *C. difficile* colonization on the day before challenge. 1 × 10^6^ CFU of *C. difficile* R20291 spores were administered orogastrically and 4 mg/kg of clindamycin was injected intraperitoneally. 2 days post infection, all animals were sacrificed. Serum and organs were extracted for downstream analysis. Serum samples were stored at -80°C prior to use. For survival rate analysis, following oral challenge, mice were monitored daily for a total of 5 days for diarrhea and other signs of disease, and moribund animals were euthanized.

### Cytokine and Chemokine Measurement

The concentrations of gastrointestinal lavage (GAL) cytokines and chemokines were measured by DuoSet^®^ ELISA Development system (R&D Systems, Minneapolis, MN, United States) according to the manufacturer’s instruction. Absorbance was measured at 450 nm using iMark^TM^ Microplate Reader (Bio-Rad, Hercules, CA, United States). Samples were measured in triplicate and statistical analyses were performed using GraphPad Prism version 6.0.

### Fecal Colony Forming Unit Determination

Fecal samples (premixed in PBS) were collected from animals and immediately subjected to heat treatment at 65°C for 20 min and then serially diluted onto BHI agar containing 0.1% taurocholate. Plates were incubated anaerobically at 37°C for 48 h and colonies were counted for CFU determination.

### RNA Extraction and Real-Time Quantitative Reverse Transcription PCR (qRT-PCR)

The colon samples were extracted with RNeasy^®^ Plus Mini kit (QIAGEN, Venlo, Netherlands). RNA yield and quality were checked by NanoDrop Spectrophotometer (Thermo Scientific, Waltham, MA, United States). Reverse transcription was performed with SuperScript^TM^ II Reverse Transcriptase (Invitrogen, Waltham, MA, United States). The expression level of proinflammatory cytokines and chemokines were measured by quantitative reverse transcription-polymerase chain reaction using RealQ Plus 2X Master Mix Green (Ampliqon, Denmark) with β-actin as the reference gene in each reaction (**Table 1**). The data were analyzed by using the ΔΔCt method and expressed as the fold change in transcript level under the test condition compared to the average for the indicated control and then normalized to the reference gene β-actin. Statistical analyses were done by using GraphPad Prism 6.0.

### Statistics

All data were expressed as the mean ± standard deviations and statistical comparisons among the groups were analyzed by Student’s *t*-test. Multiple intergroup comparisons were assessed by one-way ANOVA, followed by *post hoc* Tukey’s test with GraphPad Prism version 6.0. Statistical significance was set at *P* < 0.05.

## Results

### Preparation of Recombinant rTcdB and Nanoparticle Vaccine

**FIGURE 1:**
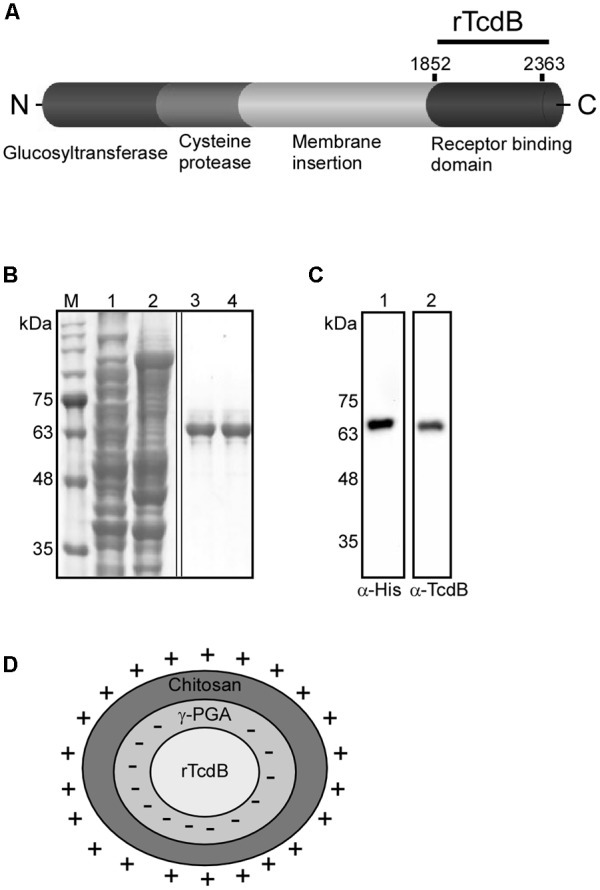
Purification of recombinant TcdB and the composition of NP-rTcdBs. **(A)** Functional domains of TcdB and regions included for nanoparticle vaccine construction. **(B)** Coomassie blue staining of purified rTcdB. rTcdB were purified from *E. coli* by His-tag affinity chromatography. Lanes 1 and 2: Total *E. coli* lysates containing rTcdB and unbound proteins. Lanes 3 and 4: Purified rTcdB with. **(C)** Immunoblotting confirmation of rTcdB. M. Protein markers. rTcdB from SDS-PAGE was transferred to a membrane and detected by anti-His (lane 1) and anti-TcdB (lane 2). **(D)** Composition of NP-rTcdBs. The ionized chitosan and γ–PGA were able to form polyelectrolyte complexes via electrostatic interactions, resulting in a matrix structure with a spherical shape.

As the immunogenicity of TcdB has been well studied, in order to evaluate the potential of using nanoparticles as *C. difficile* vaccine adjuvants, our vaccine design started with the expression of recombinant *C. difficile* toxin B RBD. The RBD of TcdB is non-toxic, have been used in other vaccine studies in the past, and is relatively easy to express and purify when compared to the full length toxin (Wang et al., 2012; Baliban et al., 2014; Spencer et al., 2014; Huang et al., 2015). The fragment comprised only the C-terminal domain region (rTcdB, amino acids 1852–2363) to avoid cytotoxicity (**Figure 1A**). PCR products were cloned into pET-21b and then transformed into *E. coli*. rTcdB was expressed in *E. coli* and purified from soluble extracts by Ni2+-NTA affinity chromatography (**Figure 1B**). Proteins were further purified to homogeneity by size-exclusion chromatography. The identity of the recombinant proteins were confirmed by Western blot analysis using Anti-His and Anti-TcdB antibody (**Figure 1C**). The overall purification yield from *E. coli* extracts was 4.5–5.0 mg/L of *E. coli* culture. His-tag of purified rTcdB was removed by thrombin cleavage.

Nanoparticles were produced using an electro-kinetic approach involving the ionic attraction of chitosan (containing positively charged –NH3 group) and γ-PGA (containing negatively charged –C00- group) which are both FDA-approved biodegradable polymers. Specific procedures for the encapsulation of rTcdB are described in the Section “Materials and Methods.” As the size of nanoparticles might influence the immunogenicity of the vaccine, we generated four different rTcdB-encapsulated nanoparticles (NP-rTcdB) with the designation NP_200_, NP_350_, NP_500_, and NP_750_ with each type of particles having a mean particle diameter of 200, 350, 500, and 750 nm, respectively. All NPs, regardless of size, contained same amount of antigen.

### Immunogenicity of Different Sizes of Nanoparticle Vaccine

**FIGURE 2:**
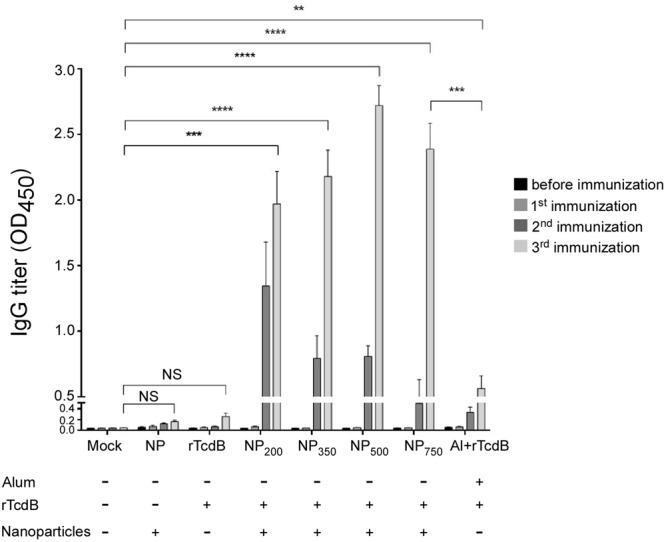
Induction of antigen-specific IgG in nanoparticle vaccinated mice. ELISA was performed in triplicate using coated peptide pool. Serum IgGs (1:20,000 dilution) in differentially immunized mice were compared. Mock: PBS injection only; NP: empty nanoparticle infection; rTcdB: injection with 20 μg purified rTcdB in PBS. NP_200_, NP_350_, NP_500_, and NP_750_: injection with NP-rTcdBs with the corresponding size in nanometers; Al+rTcdB: injection with 20 μg purified rTcdB mixed with Alum. All data are presented as mean ± standard deviations and statistical comparisons among groups were analyzed by Student’s *t*-test and ANOVA (^∗∗^*p* ≤ 0.01, ^∗∗∗^*p* ≤ 0.001, ^∗∗∗∗^*p* ≤ 0.0001). NS, not significant. All data are representative of at least three independent experiments.

To evaluate the immunogenicity of rTcdB-encapsulated nanoparticles, mice were vaccinated three times intraperitoneally with prepared nanoparticle vaccines. Sera were collected from mice before primary immunization and 1 week after each booster. No significant rTcdB specific IgGs were detected in mice injected with empty nanoparticles as expected, while IgGs were not detected in mice injected with purified recombinant rTcdB until 1 week after the third immunization (**Figure 2**). In contrast, significant IgGs were detected as earlier as 1 week after the second immunization for all NP-rTcdB immunized mice compared to the control groups (**Figure 2**). Immunization with Alum-mixed rTcdB induced detectable IgG responses albeit at a significantly lower level compared to NP-rTcdB injected groups. In terms of differences in nanoparticle sizes, while mice vaccinated with NP_200_ appeared to have higher IgGs after the first injection, NP_500_ and NP_750_ elicited a higher level of IgG with subsequent boosters. The highest IgG level detected for all sizes of nanoparticles were after the third immunization.

**FIGURE 3:**
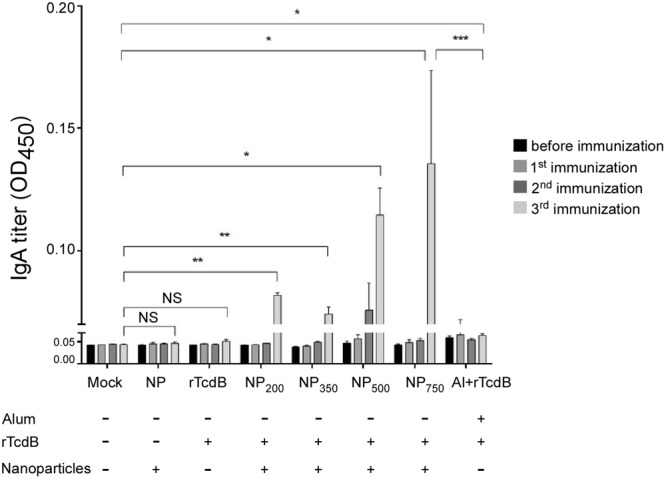
Induction of antigen-specific IgA in nanoparticle vaccinated mice. ELISA was performed in triplicate using coated peptide pool. Serum IgAs (1:1,000 dilution) in differentially immunized mice were compared (*n* = 5 per group). Mock: PBS injection only; NP: empty nanoparticle infection; rTcdB: injection with 20 μg purified rTcdB in PBS. NP_200_, NP_350_, NP_500_, and NP_750_: injection with NP-rTcdBs with the corresponding size in nanometers; Al+rTcdB: injection with 20 μg purified rTcdB mixed with Alum. All data are presented as mean ± standard deviations and statistical comparisons among groups were analyzed by Student’s *t*-test and ANOVA (*n* = 5, ^∗^*p* ≤ 0.05, ^∗∗^*p* ≤ 0.01, ^∗∗∗^*p* ≤ 0.001). NS, not significant. All data are representative of at least three independent experiments.

On the other hand, rTcdB-specific IgAs were observed only after the third immunization (**Figure 3**). All four sizes of nanoparticle vaccines induced a detectable level of IgAs with a general trend toward larger NPs inducing higher antibody responses. Only baseline titers were observed in empty NPs immunized control, purified rTcdB immunized control, and the PBS control groups. Alum-mixed rTcdB induced little to no IgAs. Taken together, these results showed that vaccination with NP-rTcdB was much more effective in inducing antigen-specific IgG and IgA than the traditional aluminum hydroxide adjuvant.

**FIGURE 4:**
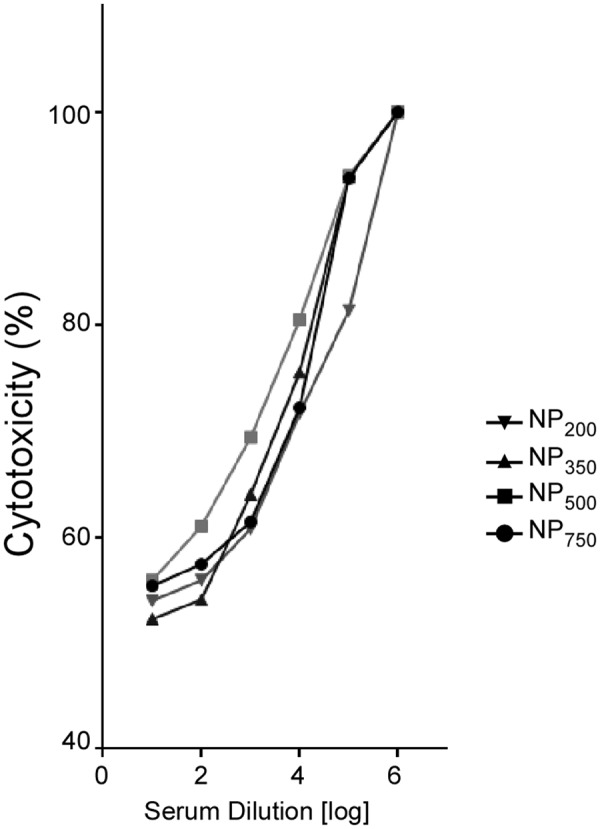
*In vitro* evaluation of antibody-mediated neutralization of TcdB. Serum samples from immunized mice (*n* = 5) were incubated with purified TcdB and then mixed with Vero cells. Cytotoxicity was assayed by lactate dehydrogenase (LDH) assay. No significant differences were detected between each immunized group. All data are representative of three independent experiments.

To determine the functional capacity of anti-rTcdB antibodies induced by our nanoparticle vaccine to neutralize native toxin B, neutralizing efficacy was assessed against purified toxin B (**Figure 4**). After incubation of Vero cells with serial dilutions of serum, the cells were assayed for the release of lactate dehydrogenase (LDH). Antibodies raised against nanoparticle vaccines were found to have neutralizing activity in a dose-dependent manner and no significant differences were observed between all four NP sizes (**Figure 4**). Control animals that received adjuvant alone or rTcdB alone did not produce sufficient antibodies to neutralize the cytotoxic effect of toxin B (data not shown).

### Persistence of the Nanoparticle Vaccine-Induced Antibodies

**FIGURE 5:**
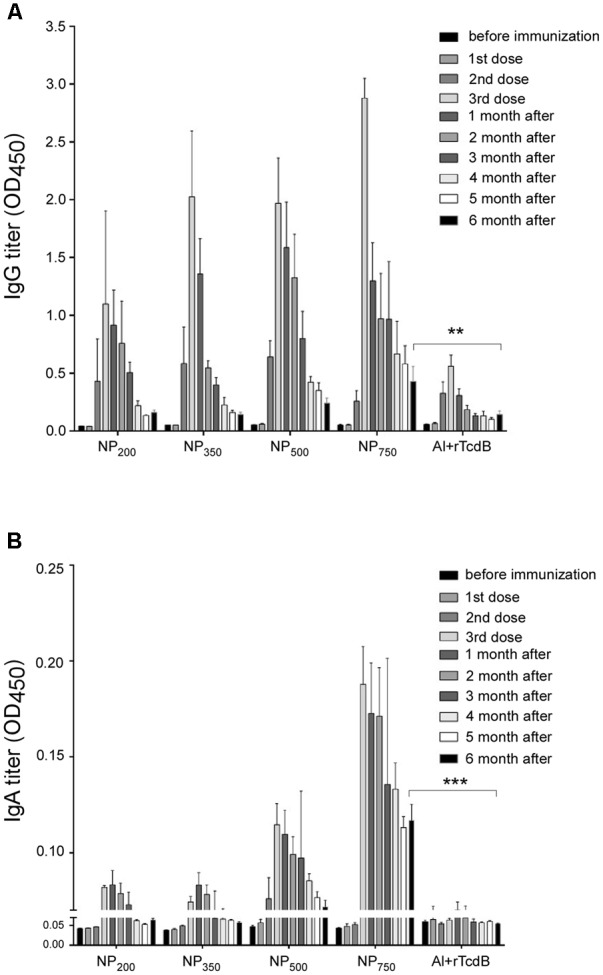
Long-term antibody response of immunized mice. rTcdB-specific IgG (1:20,000 dilution) **(A)** and IgA (1: 1,000 dilution) **(B)** titers from the serum of immunized mice were determined by ELISA. NP_200_, NP_350_, NP_500_, NP_750_: injection with NP-rTcdBs with the corresponding size in nanometers; Al+rTcdB: injection with 20 μg purified rTcdB mixed with Alum. All data are presented as mean ± standard deviations and statistical comparisons among groups were analyzed by Student’s *t*-test and ANOVA (*n* = 5, ^∗∗^*P* ≤ 0.01, ^∗∗∗^*p* ≤ 0.001, ^∗∗∗∗^*p* ≤ 0.0001). NS, not significant. All data are representative of at least three independent experiments.

To further evaluate the potency of various sizes of NP-rTcdB as vaccine candidates, serum antibody responses of immunized mice were measured for up to 6 months after the last immunization. As shown in **Figures 5A,B**, both IgG and IgA levels detected in all NP-rTcdB vaccinated mice peaked after the third immunization and were still detectable 6 months after the last immunization (**Figure 5**). Antibody titer for NP_750_ vaccinated mice at 6-month after immunization were either not significantly different (IgGs) or higher (IgAs) than those that were immunized with alum mixed rTcdB (*P* < 0.05). In terms of nanoparticle size, IgG titer of NP_750_ immunized mice at 6-month post-immunization were significantly higher than mice immunized with smaller size nanoparticle vaccines (*P* < 0.05).

### Protective Efficacy of Nanoparticle Vaccine

**FIGURE 6:**
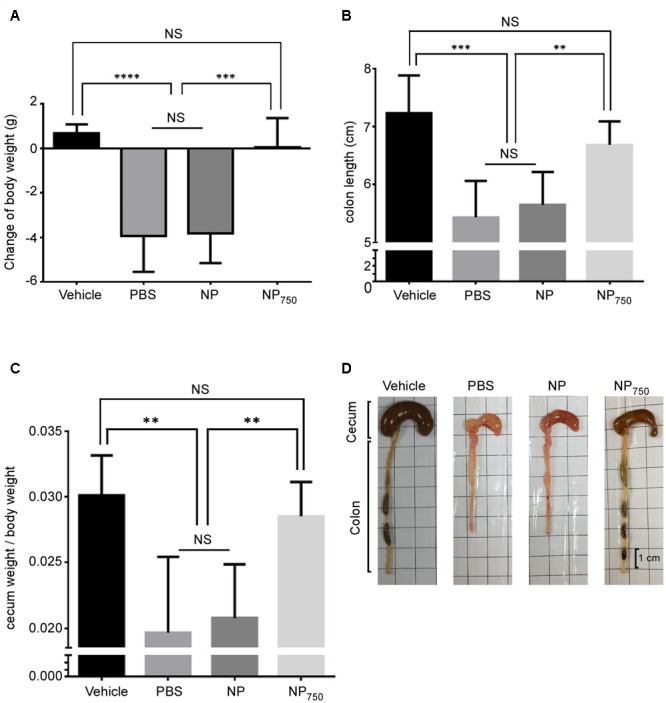
Protection against purified *C. difficile* spores challenge in vaccinated mice. Various groups of mice were treated with an antibiotic cocktail and then challenged by *C. difficile* or PBS only for 2 days. Body weight change **(A)**, colon length **(B)**, cecum weight **(C)**, and gross views of cecum and colon **(D)** were assessed. Vehicle: non-immunized and non-infected group; PBS: mock-immunized and infected group; NP: empty nanoparticle immunized and infected group; NP_750_: vaccine immunized and infected group. All data are representative of at least three independent experiments. All data are presented as mean ± standard deviations and statistical comparisons among groups were analyzed by Student’s t-test (^∗∗^*p* ≤ 0.01, ^∗∗∗^*p* ≤ 0.001). NS = not significant.

**FIGURE 7:**
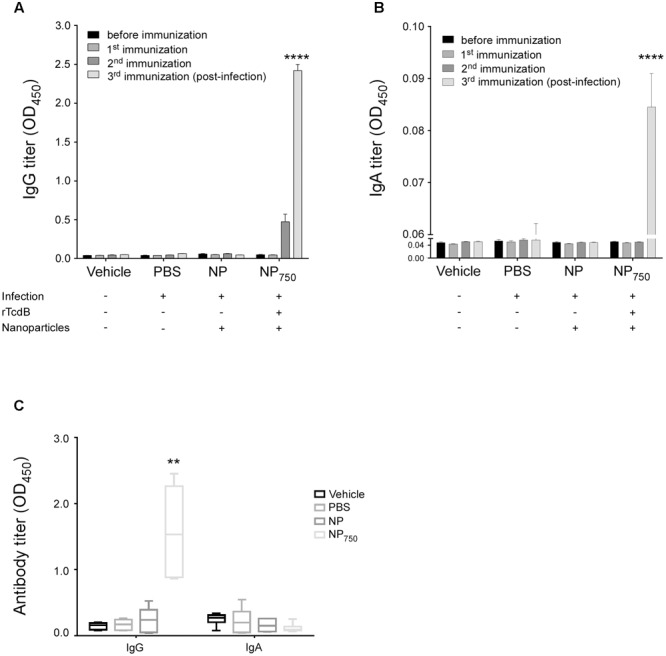
Presence of antigen-specific IgG and IgA in NP-rTcdB vaccinated mice after *C. difficile* challenge. rTcdB-specific IgG (1:20,000 dilution) **(A)** and IgA (1: 1,000 dilution) **(B)** from serum of different treatment groups were compared by ELISA. **(C)** rTcdB-specific IgG and IgA from gastrointestinal lavage (GAL) (1:2 dilution) were also compared. All data are presented as mean ± standard deviations. Statistical comparisons between NP-rTcdB vaccinated and empty nanoparticle vaccinated group were analyzed by Student’s *t*-test (^∗∗^*P* ≤ 0.01, ^∗∗∗^*p* ≤ 0.001, ^∗∗∗∗^*p* ≤ 0.0001). All data are representative of at least three independent experiments.

In order to determine the protective efficacy of immunization with nanoparticle vaccines upon challenge with *C. difficile* spores. The high-toxin producing *C. difficile* strain R20291 was used for infection. We immunized C57BL/6 mice by i.p. injection with NP_750_ three times. Control groups include mice immunized with PBS alone, and empty nanoparticle alone. After the third immunization, the normal gut microbiota of immunized mice was perturbed with antibiotic cocktails dissolved in the drinking water for 5 days followed by a single i.p. injection of clindamycin prior to challenge with purified R20291 *C. difficile* spores (5 × 10^5^ spores). Infected mice in the control group receiving either PBS or empty nanoparticle (NP) showed signs of CDI, including loss of body and cecal weight and a decrease in colon length (**Figures 6A–C**). Gross view of colon and cecum indicated acute stage of colitis (**Figure 6D**). In contrast, NP_750_ immunized animal displayed no body weight loss and cecum weight and colon length were similar to that of the healthy control (Vehicle). Gross view of colon and cecum from NP_750_ vaccinated mice also were similar in appearance to the healthy control with visibly formed fecal samples being retained in the colon (**Figure 6D**). Serum samples obtained before and post-infection were obtained and evaluated by ELISA to assess the development of specific antibody response. Serum from NP_750_ immunized group induced significant serum IgG and IgA responses post infection, while only baseline titers were detected from the PBS and NP control group (**Figures 7A,B**). In addition to systemic antibody response, the GAL were collected from each mouse to test for intestinal antigen-specific antibody responses. As shown in **Figure 7C**, significantly higher IgG responses were detected from GAL samples of NP_750_ immunized mice compared to control groups. As expected, no detectable IgAs were observed in samples from NP100 group since the vaccine was delivered intraperitoneally. To assess whether immunization with NP-rTcdBs would also influence *C. difficile* replication, fecal samples were collected from animals 2 days post infection. As shown in Supplementary Figure 1, no significant differences were observed between all groups.

**FIGURE 8:**
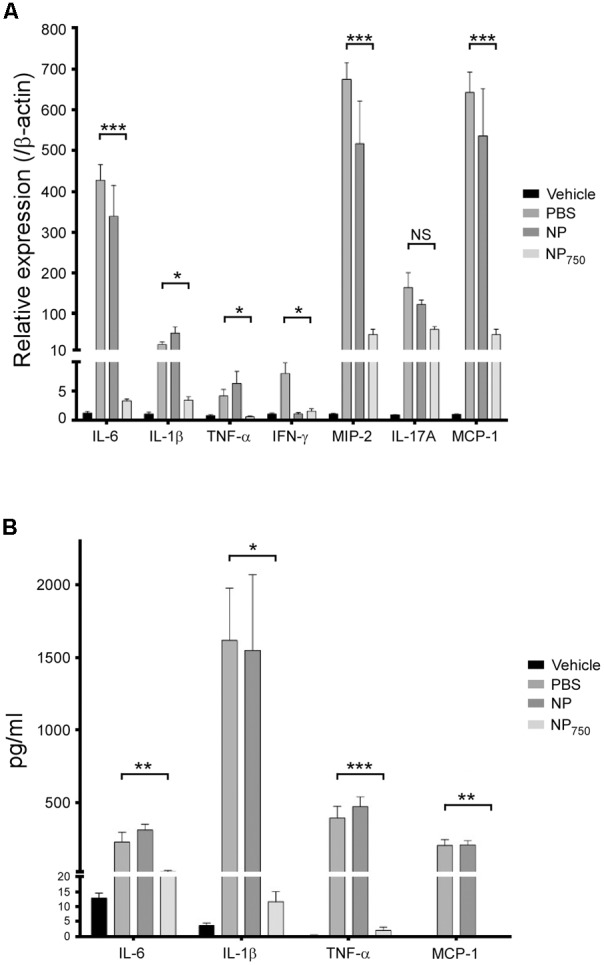
Decreased level of proinflammatory cytokines in *C. difficile*-infected mice vaccinated by NP-rTcdB. The level of various proinflammatory cytokines and chemokines in colon tissues **(A)** and GAL **(B)** of vehicle group, mock-vaccinated group (PBS), empty nanoparticle-vaccinated group, and NP_750_ vaccinated group as measured by real-time polymerase chain reactions and ELISA, respectively. All data are presented as mean ± standard deviations and statistical comparisons among groups were analyzed by Student’s *t*-test (^∗^*p* ≤ 0.05, ^∗∗^*p* ≤ 0.01, ^∗∗∗^*p* ≤ 0.001, ^∗∗∗∗^*p* ≤ 0.0001). NS, not significant. All data are representative of at least three independent experiments.

Consistent with the results observed above, the expression of proinflammatory cytokines interleukin 6 (IL-6), interleukin 1β (IL-1β), tumor necrosis factor α (TNF-α), interferon γ (IFN-γ), macrophage inflammatory protein 2 (MIP-2), monocyte chemoattractant protein 1 (MCP-1), and interleukin 17A (IL-17A), was significantly increased in colons of the control group mice vaccinated with PBS or empty nanoparticle compared to healthy control (**Figure 8A**). In contrast, colons from the NP_750_ immunized group contained significantly lower level of inflammatory cytokines and chemokines level with the exception of IL-17A (**Figure 8A**). In addition, proinflammatory cytokines and chemokines within GAL were measured by ELISA (**Figure 8B**). The level of IL-6, IL-1β, TNF-α, and MCP-1 was increased in GAL of mice immunized with PBS or empty nanoparticles compared to the healthy control. However, mice immunized with NP_750_ displayed significant decrease level of cytokines and chemokines tested (**Figure 8B**).

**FIGURE 9:**
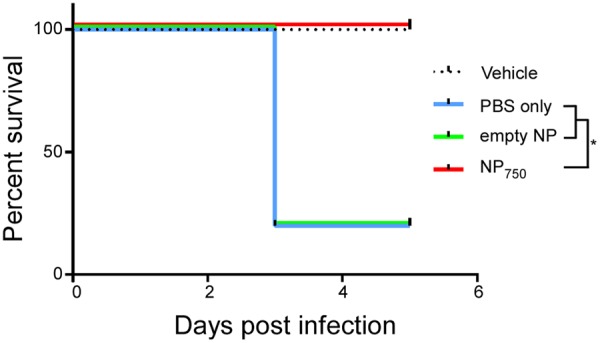
Complete protection against *C. difficile* challenge in vaccinated mice. Various groups of mice were treated with an antibiotic cocktail and then challenged by *C. difficile* or PBS. Survival was monitored for 5 days. Vehicle: health control; PBS: infection control; NP: empty nanoparticle vaccination; NP_750_: NP-rTcdB vaccination. All data are representative of at least three independent experiments (^∗^*p* ≤ 0.05, error bars indicate standard errors of the means; the data were analyzed by Kaplan–Meier survival analysis).

Finally, the survival rate of i.p. immunized animal post infection was recorded for up to 6 days post-infection (**Figure 9**). Eighty of mice in control groups vaccinated with PBS or empty nanoparticle were susceptible to *C. difficile* infection and died on day 3 post infection. In contrast, all mice vaccinated with NP_750_ were completely protected against the lethal *C. difficile* spore challenge (*P* < 0.001). In short, these results suggest that immunization with nanoparticle vaccines can protect mice from severe CDI.

## Discussion

*Clostridium difficile* is one of the major cause of worldwide infectious diarrhea in healthcare systems (Ananthakrishnan, 2011). Vaccine development has been considered by many to be one way to control the morbidity and relapses due to CDI (Martin and Wilcox, 2016). Most studies on the development of vaccines against CDI focused on the major pathogenic determinants of *C. difficile*, toxin A and B (Martin and Wilcox, 2016). Production of anti-toxin antibodies are considered to be the most effective defense mechanism in mediating systemic and mucosal protection against CDI in both animal models and patients (Pechine and Collignon, 2016). In recent years, numerous studies have reported an increase in the prevalence of TcdA- TcdB+ isolates while *in vivo* evidence demonstrated that such toxigenic strain is fully virulent in hamsters (Alfa et al., 2000; Kuijper et al., 2001; Drudy et al., 2007; Carter et al., 2015). Clinically, TcdA-TcdB+ isolates have been found to cause the entire symptoms of CDI and *in vitro* studies have shown TcdB to be more potent than TcdA in causing human colonic tissue necrosis and decreasing barrier function (Hensgens et al., 2012; Eyre et al., 2013; Lim et al., 2014). Studies have shown that the critical antigenic determinants of toxins B are localized to the repetitive oligopeptides contained within the C-terminal and can induce neutralizing antibody responses (Wang et al., 2012; Baliban et al., 2014; Huang et al., 2015). Equally important in the development of an effective vaccine is adjuvant selection. In this study, we decided to focus on using the receptor domain (residues 1852–2363) of TcdB as a first test of evaluating the potential of using biodegradable nanoparticles as an encapsulating adjuvant.

The polyelectrolyte complex nanoparticles used in this study was formed by the ionic interactions between two oppositely charged polymers, chitosan (containing positively charged –NH3+) and γ-PGA (containing negatively charged –COO–) (**Figure 1D**). We were successful in encapsulating the purified rTcdB with these two components to form double-layered nanoparticles with sizes ranging from 200 to 750 nm in diameter. Surface charge is one of the most important factors affecting the function of nanoparticles in adhering and transporting across the intestinal epithelial cells (Frohlich, 2012; Feng et al., 2015). Positively charged nanoparticles are easier to be taken up by cells than negatively charged ones (Chen L. et al., 2011; Yue et al., 2011). The mechanism by which nanoparticles are transported across the epithelium is still not very well understood. It is probably due to the interaction between the positively charged amino group of the nanoparticles and the negatively charged site on the luminal aspect-oriented epithelial cells. The tight junction protein ZO-1 and F-actin are redistributed which is accompanied by an increase in paracellular permeability and the opening of the tight junction (Ballard et al., 1995; Kotze et al., 1998; Conner and Schmid, 2003; Lin et al., 2007). The other hypothesis of transcellular transport is by nanoparticle absorption (Conner and Schmid, 2003). The hydrophobic nature of nanoparticles helped in creating stronger attachments to anionic cell membrane due to electrostatic interaction, which can result in the transport of the particles across the cells and eventual release at the basolateral pole (Chen M.C. et al., 2011). Moreover, it is known that positively charged nanoparticles can increase CD4+ T-cell activation and germinal center B-cell expansion in the local lymph nodes. Therefore, in the design of our nanoparticle vaccine, the positively charged chitosan was incorporated as the outer layer. Aside from surface charges, particle size also plays an important role in deciding the cellular localization of polymer particles (Sahoo et al., 2002). Several studies indicated that as particle size decreases, the corresponding antibody responses also diminishes (Giuliano et al., 2002; Gutierro et al., 2002; Katare et al., 2005; Kanchan and Panda, 2007). In our experimental data, the larger NPs indeed elicited higher rTcdB-specific antibody responses, and the highest titer of IgG and IgA were obtained with NPs having a diameter of about 750 nm (NP_750_). Furthermore, 6 months after the final booster injection, groups that received NP_750_ retained significantly more IgGs and IgAs compared to other NPS. To induce antibody production using extracellular antigens, it is essential that the peptide fragment of the antigen binds to the MHC II molecule in the plasma membrane of APCs (Trombetta and Mellman, 2005). As particle size decreases, the available surface area of the antigen-loaded particles for attachment may also decrease which can result in lower antibody response. A second possibility to explain our observation is that while virus-sized particles (20–200 nm) are usually taken up by cells via endocytosis, which results in T-helper type 1 humoral immune response, the larger size NPs (>500 nm) are mainly taken up via phagocytosis and are more likely to promote a T-helper type 2 humoral immune response (Xiang et al., 2006). Additional studies such T lymphocyte proliferation assays are currently underway in our laboratory to characterize in depth the immune responses elicited by the different sized NPs.

The role of the adaptive immunity in the outcome of *C. difficile* colonization and disease progression has been appreciated for many years (Mulligan et al., 1993; Kyne et al., 2000). Initial challenge with *C. difficile* can stimulate IL-10 and IL-4 which in turn stimulate the maturation of naïve B cells and subsequence immunoglobulin production (Ryan et al., 2011). An increase in the level of both toxin-specific IgA and IgG responses have been linked to a decrease in chance of having recurrent CDIs (Kyne et al., 2001). In our study, after three rounds of immunization by i.p. injection higher and longer-lasting titers of antigen-specific IgGs were observed when mice were given NP-rTcdB as compared to rTcdB premixed with aluminum hydroxide. Likewise, significant IgAs were also detected after NP-rTcdB vaccination. Furthermore, regardless the size of the nanoparticles, the nanoparticle vaccines were able to induce significant antigen-specific IgGs and IgAs, and these antibodies displayed effective toxin B neutralization activity *in vitro*.

In evaluating the protection afforded by immunization with our nanoparticle vaccine, we observed that immunization with nanoparticle vaccine prior to *C. difficile* infection diminished symptoms of CDI as demonstrated by lower body and cecal weight loss, longer colon, lack of diarrhea, a general healthier cecum and colon morphology, and decrease in the level of colonic and GAL inflammatory cytokine and chemokine when compared to mice immunized with PBS or empty nanoparticles. Importantly, immunization with NP100 provided mice 100% protection from lethal spore challenge as compared to an average of 80% mortality in mice immunized with PBS or empty nanoparticles. Lastly, we determined the level of rTcdB-specific antibodies in vaccinated mice monthly and observed significant antibody level at 6 months after immunization. Antibody responses of mice vaccinated with nanoparticles were longer lasting than those generated from mice vaccinated with aluminum hydroxide mixed rTcdB even when the level of the antigen used in the former was five times lesser than used in the latter (20 μg vs. 100 μg). However, further studies will be required to determine whether these long-lasting antibodies can translate into longer-lasting protection.

The level of serum IgG antibodies against both TcdA and TcdB have been shown to be correlated with protection against CDI (Zhao S. et al., 2014; Kociolek and Gerding, 2016). An early study involving human patients observed correlation between clinical recovery with no relapse with higher TcdB IgG titers. In hospitalized patients, asymptomatic *C. difficile* carriers were found to have significantly higher serum IgG antibody levels compared to those who developed diarrhea (Kyne et al., 2000; Zhao S. et al., 2014). Less is known about the role of serum IgA responses. Johnson et al. (1995) found that serum IgA from patients were able to neutralize the effect of TcdA. The importance of serum IgA against TcdB in resolving CDI remains unclear and will require further investigation. In addition to serum anti-toxin antibody responses, activated DCs will promote a Th2 response which will induce mucosal specific adaptive immunity. Warny et al. (1994) showed that fecal IgA antibody titers were significantly higher in patients who had only single episode of CDI compared to those who relapsed. Similarly, lower level of fecal and colonic IgAs have been shown to correlate with extended diseases and recurrences (Johal et al., 2004; Pechine and Collignon, 2016). In our study, although significant induction of mucosal IgGs were observed in colonic lavage fluids for the group that received nanoparticle vaccination, no significant induction of mucosal IgAs were found in colon lavage fluids. We hypothesize this was due to vaccine being delivered intraperitoneally rather than through the mucosal route. The limit of i.p. delivery has also prompted us to begun to evaluate the feasibility of supplying our nanoparticle vaccine via orogastricdelivery. Interestingly, secretory IgAs was detected in colonic lavage fluids and studies are underway to understand whether these antibodies have protective roles (unpublished data).

Since the discovery of *C. difficile* as the major causative agent of antibiotic associated diarrhea, the role of TcdA and TcdB in the underlying disease mechanisms has been well studied. Similarly, numerous vaccine studies have also been initiated. Currently, three vaccines against CDI are being tested in clinical trials (Kociolek and Gerding, 2016). The most advanced being the toxoid vaccine originally developed by Acambis (ACAM-CDIFFTM) and now being developed and tested by Sanofi-Pasteur (CDIFFENSE^TM^) (Sougioultzis et al., 2005; Foglia et al., 2012). The intramuscularly delivered vaccine contains formalin inactivated TcdA and TcdB adjuvanted with alum. Both Phase I and Phase II studies have been completed and the vaccine appear to be safe and immunogenic with no adverse events reported. Concerns has been raised for possible residual toxicity associated with formalin inactivation as well as the observation that the vaccine might not be active for weeks to months even after a regimen of three administrations (Zhao S. et al., 2014; Kociolek and Gerding, 2016). A vaccine developed by Pfizer using genetically modified full length TcdA and TcdB have completed phase II testing and phase III begun this year (Tian et al., 2012). VLA84, a recombinant vaccine consisting of truncated TcdA fused to TcdB has completed phase I study (Bezay et al., 2016). Although results from these clinical trials have generally been positive, but the long term protection afforded by these vaccine remains unknown. Since these vaccines targets only toxins, colonization of *C. difficile* in the gastrointestinal tract is not expected to be affected (Kociolek and Gerding, 2016). Similarly, our own study is also limited by using truncated TcdB as the sole antigen, which was shown to have no effect on *C. difficile* colonization. However, the advantages of our strategy in using nanoparticle as vaccine adjuvant are the general safety of the biodegradable material, low production cost, and rapid encapsulation. We have begun to test the effectiveness of encapsulating both toxin fragments as well as surface proteins. Furthermore, the advantage of nanoparticle vaccine is the possibility of inducing mucosal immunity via oral or nasal delivery, which is currently being tested in our lab.

Collectively, results generated from this study suggested that the receptor domain of toxin B encapsulated by a biodegradable chitosan/γ-PGA based nanocomplex can elicit strong antigen specific antibody response when given intraperitoneally. Furthermore, such immune response can protect immunized mice from lethal challenge of *C. difficile* spores. Future work will focus on testing the long-term protection potential of the nanoparticle vaccine, and whether protection can be extended to infection by other *C. difficile* clinical isolates. In conclusion, this study demonstrated that nanoparticle-based vaccine may be used as a safe and effective vaccine adjuvant against CDI.

## Author Contributions

Y-WL, J-WC, P-JT, and I-HH designed the experiments. Y-WL and Y-HC carried out the experiments, Y-WL, J-WC, and I-HH analyzed the data. Y-WL, J-WC, and I-HH prepared the manuscript.

## Conflict of Interest Statement

The authors declare that the research was conducted in the absence of any commercial or financial relationships that could be construed as a potential conflict of interest.

## References

[B1] Alfa M. J. Kabani A. Lyerly D. Moncrief S. Neville L. M. Al-Barrak A. (2000). Characterization of a toxin A-negative, toxin B-positive strain of *Clostridium difficile* responsible for a nosocomial outbreak of *Clostridium difficile*-associated diarrhea. *J. Clin. Microbiol.* 38 2706–2714. 10878068 10.1128/jcm.38.7.2706-2714.2000 PMC87004

[B2] Ananthakrishnan A. N. (2011). *Clostridium difficile* infection: epidemiology, risk factors and management. *Nat. Rev. Gastroenterol. Hepatol.* 8 17–26. 10.1038/nrgastro.2010.190 21119612

[B3] Aslam S. Musher D. M. (2006). An update on diagnosis, treatment, and prevention of *Clostridium difficile*-associated disease. *Gastroenterol. Clin. North Am.* 35 315–335. 10.1016/j.gtc.2006.03.009 16880068

[B4] Awad M. M. Johanesen P. A. Carter G. P. Rose E. Lyras D. (2014). *Clostridium difficile* virulence factors: insights into an anaerobic spore-forming pathogen. *Gut Microbes* 5 579–593. 10.4161/19490976.2014.969632 25483328 PMC4615314

[B5] Baliban S. M. Michael A. Shammassian B. Mudakha S. Khan A. S. Cocklin S. (2014). An optimized, synthetic DNA vaccine encoding the toxin A and toxin B receptor binding domains of *Clostridium difficile* induces protective antibody responses in vivo. *Infect. Immun.* 82 4080–4091. 10.1128/IAI.01950-14 25024365 PMC4187890

[B6] Ballard S. T. Hunter J. H. Taylor A. E. (1995). Regulation of tight-junction permeability during nutrient absorption across the intestinal epithelium. *Annu. Rev. Nutr.* 15 35–55. 10.1146/annurev.nu.15.070195.000343 8527224

[B7] Bartlett J. G. (2008). Historical perspectives on studies of *Clostridium difficile* and *C. difficile* infection. *Clin. Infect. Dis.* 46(Suppl. 1) S4–S11. 10.1086/521865 18177220

[B8] Bezay N. Ayad A. Dubischar K. Firbas C. Hochreiter R. Kiermayr S. (2016). Safety, immunogenicity and dose response of VLA84, a new vaccine candidate against *Clostridium difficile*, in healthy volunteers. *Vaccine* 34 2585–2592. 10.1016/j.vaccine.2016.03.098 27079932

[B9] Borgia G. Maraolo A. E. Foggia M. Buonomo A. R. Gentile I. (2015). Fecal microbiota transplantation for *Clostridium difficile* infection: back to the future. *Expert Opin. Biol. Ther.* 15 1001–1014. 10.1517/14712598.2015.1045872 26063385

[B10] Braun V. Hundsberger T. Leukel P. Sauerborn M. von Eichel-Streiber C. (1996). Definition of the single integration site of the pathogenicity locus in *Clostridium difficile*. *Gene* 181 29–38. 8973304 10.1016/s0378-1119(96)00398-8

[B11] Carter G. P. Chakravorty A. Pham Nguyen T. A. Mileto S. Schreiber F. Li L. (2015). Defining the roles of TcdA and TcdB in localized gastrointestinal disease, systemic organ damage, and the host response during *Clostridium difficile* infections. *mBio* 6:e00551-15 10.1128/mBio.00551-15 PMC4453007 26037121

[B12] Carter G. P. Rood J. I. Lyras D. (2012). The role of toxin A and toxin B in the virulence of *Clostridium difficile*. *Trends Microbiol.* 20 21–29. 10.1016/j.tim.2011.11.003 22154163

[B13] Chen L. McCrate J. M. Lee J. C. Li H. (2011). The role of surface charge on the uptake and biocompatibility of hydroxyapatite nanoparticles with osteoblast cells. *Nanotechnology* 22:105708 10.1088/0957-4484/22/10/105708 PMC3144725 21289408

[B14] Chen M. C. Sonaje K. Chen K. J. Sung H. W. (2011). A review of the prospects for polymeric nanoparticle platforms in oral insulin delivery. *Biomaterials* 32 9826–9838. 10.1016/j.biomaterials.2011.08.087 21925726

[B15] Conner S. D. Schmid S. L. (2003). Regulated portals of entry into the cell. *Nature* 422 37–44. 10.1038/nature01451 12621426

[B16] Di Bella S. Ascenzi P. Siarakas S. Petrosillo N. di Masi A. (2016). *Clostridium difficile* toxins A and B: insights into pathogenic properties and extraintestinal effects. *Toxins* 8:E134 10.3390/toxins8050134 PMC4885049 27153087

[B17] Dobson G. Hickey C. Trinder J. (2003). *Clostridium difficile* colitis causing toxic megacolon, severe sepsis and multiple organ dysfunction syndrome. *Intensive Care Med.* 29 1030 10.1007/s00134-003-1754-7 12734650

[B18] Drudy D. Fanning S. Kyne L. (2007). Toxin A-negative, toxin B-positive *Clostridium difficile*. *Int. J. Infect. Dis.* 11 5–10. 10.1016/j.ijid.2006.04.003 16857405

[B19] Eyre D. W. Cule M. L. Wilson D. J. Griffiths D. Vaughan A. O’Connor L. (2013). Diverse sources of *C. difficile* infection identified on whole-genome sequencing. *N. Engl. J. Med.* 369 1195–1205. 10.1056/NEJMoa1216064 24066741 PMC3868928

[B20] Feng C. Li J. Kong M. Liu Y. Cheng X. J. Li Y. (2015). Surface charge effect on mucoadhesion of chitosan based nanogels for local anti-colorectal cancer drug delivery. *Colloids Surf. B Biointerfaces* 128 439–447. 10.1016/j.colsurfb.2015.02.042 25769283

[B21] Foglia G. Shah S. Luxemburger C. Pietrobon P. J. (2012). *Clostridium difficile*: development of a novel candidate vaccine. *Vaccine* 30 4307–4309. 10.1016/j.vaccine.2012.01.056 22682287

[B22] Frohlich E. (2012). The role of surface charge in cellular uptake and cytotoxicity of medical nanoparticles. *Int. J. Nanomed.* 7 5577–5591. 10.2147/IJN.S36111 PMC3493258 23144561

[B23] Giuliano E. A. Moore C. P. Phillips T. E. (2002). Morphological evidence of M cells in healthy canine conjunctiva-associated lymphoid tissue. *Graefes Arch. Clin. Exp. Ophthalmol.* 240 220–226. 10.1007/s00417-002-0429-3 11935280

[B24] Glenny A. T. (1930). Insoluble precipitates in diphtheria and tetanus immunization. *Br. Med. J.* 2 244–245. 20775638 10.1136/bmj.2.3632.244 PMC2450349

[B25] Guo S. Yan W. McDonough S. P. Lin N. Wu K. J. He H. (2015). The recombinant *Lactococcus lactis* oral vaccine induces protection against *C. difficile* spore challenge in a mouse model. *Vaccine* 33 1586–1595. 10.1016/j.vaccine.2015.02.006 25698490

[B26] Gutierro I. Hernandez R. M. Igartua M. Gascon A. R. Pedraz J. L. (2002). Size dependent immune response after subcutaneous, oral and intranasal administration of BSA loaded nanospheres. *Vaccine* 21 67–77. 12443664 10.1016/s0264-410x(02)00435-8

[B27] Hensgens M. P. Keessen E. C. Squire M. M. Riley T. V. Koene M. G. de Boer E. (2012). Clostridium difficile infection in the community: a zoonotic disease? *Clin. Microbiol. Infect.* 18 635–645. 10.1111/j.1469-0691.2012.03853.x 22536816

[B28] Huang J. H. Wu C. W. Lien S. P. Leng C. H. Hsiao K. N. Liu S. J. (2015). Recombinant lipoprotein-based vaccine candidates against *C. difficile* infections. *J. Biomed. Sci.* 22 65 10.1186/s12929-015-0171-x PMC4527207 26245825

[B29] Hung Y. P. Ko W. C. Chou P. H. Chen Y. H. Lin H. J. Liu Y. H. (2015). Proton-pump inhibitor exposure aggravates *Clostridium difficile*-associated colitis: evidence from a mouse model. *J. Infect. Dis.* 212 654–663. 10.1093/infdis/jiv184 25805751

[B30] Johal S. S. Lambert C. P. Hammond J. James P. D. Borriello S. P. Mahida Y. R. (2004). Colonic IgA producing cells and macrophages are reduced in recurrent and non-recurrent *Clostridium difficile* associated diarrhoea. *J. Clin. Pathol.* 57 973–979. 10.1136/jcp.2003.015875 15333661 PMC1770426

[B31] Johnson S. (2009). Recurrent *Clostridium difficile* infection: a review of risk factors, treatments, and outcomes. *J. Infect.* 58 403–410. 10.1016/j.jinf.2009.03.010 19394704

[B32] Johnson S. Sambol S. P. Brazier J. S. Delmee M. Avesani V. Merrigan M. M. (2003). International typing study of toxin A-negative, toxin B-positive *Clostridium difficile* variants. *J. Clin. Microbiol.* 41 1543–1547. 12682143 10.1128/JCM.41.4.1543-1547.2003 PMC153904

[B33] Johnson S. Sypura W. D. Gerding D. N. Ewing S. L. Janoff E. N. (1995). Selective neutralization of a bacterial enterotoxin by serum immunoglobulin A in response to mucosal disease. *Infect. Immun.* 63 3166–3173. 7622244 10.1128/iai.63.8.3166-3173.1995 PMC173432

[B34] Kalam M. A. Khan A. A. Alshamsan A. (2017). Non-invasive administration of biodegradable nano-carrier vaccines. *Am. J. Transl. Res.* 9 15–35. 28123631 PMC5250701

[B35] Kanchan V. Panda A. K. (2007). Interactions of antigen-loaded polylactide particles with macrophages and their correlation with the immune response. *Biomaterials* 28 5344–5357. 10.1016/j.biomaterials.2007.08.015 17825905

[B36] Karch C. P. Burkhard P. (2016). Vaccine technologies: from whole organisms to rationally designed protein assemblies. *Biochem. Pharmacol.* 120 1–14. 10.1016/j.bcp.2016.05.001 27157411 PMC5079805

[B37] Katare Y. K. Muthukumaran T. Panda A. K. (2005). Influence of particle size, antigen load, dose and additional adjuvant on the immune response from antigen loaded PLA microparticles. *Int. J. Pharm.* 301 149–160. 10.1016/j.ijpharm.2005.05.028 16023313

[B38] Kociolek L. K. Gerding D. N. (2016). Breakthroughs in the treatment and prevention of *Clostridium difficile* infection. *Nat. Rev. Gastroenterol. Hepatol.* 13 150–160. 10.1038/nrgastro.2015.220 26860266

[B39] Kotze A. F. Luessen H. L. de Leeuw B. J. de Boer A. G. Verhoef J. C. Junginger H. E. (1998). Comparison of the effect of different chitosan salts and N-trimethyl chitosan chloride on the permeability of intestinal epithelial cells (Caco-2). *J. Control. Release* 51 35–46. 9685902 10.1016/s0168-3659(97)00154-5

[B40] Kuehne S. A. Cartman S. T. Heap J. T. Kelly M. L. Cockayne A. Minton N. P. (2010). The role of toxin A and toxin B in *Clostridium difficile* infection. *Nature* 467 711–713. 10.1038/nature09397 20844489

[B41] Kuijper E. J. de Weerdt J. Kato H. Kato N. van Dam A. P. van der Vorm E. R. (2001). Nosocomial outbreak of *Clostridium difficile*-associated diarrhoea due to a clindamycin-resistant enterotoxin A-negative strain. *Eur. J. Clin. Microbiol. Infect. Dis.* 20 528–534. 11681431 10.1007/s100960100550

[B42] Kyne L. Warny M. Qamar A. Kelly C. P. (2000). Asymptomatic carriage of *Clostridium difficile* and serum levels of IgG antibody against toxin A. *N. Engl. J. Med.* 342 390–397. 10.1056/NEJM200002103420604 10666429

[B43] Kyne L. Warny M. Qamar A. Kelly C. P. (2001). Association between antibody response to toxin A and protection against recurrent *Clostridium difficile* diarrhoea. *Lancet* 357 189–193. 10.1016/S0140-6736(00)03592-3 11213096

[B44] Leroux-Roels G. (2010). Unmet needs in modern vaccinology: adjuvants to improve the immune response. *Vaccine* 28(Suppl. 3) C25–C36. 10.1016/j.vaccine.2010.07.021 20713254

[B45] Lim S. K. Stuart R. L. Mackin K. E. Carter G. P. Kotsanas D. Francis M. J. (2014). Emergence of a ribotype 244 strain of *Clostridium difficile* associated with severe disease and related to the epidemic ribotype 027 strain. *Clin. Infect. Dis.* 58 1723–1730. 10.1093/cid/ciu203 24704722

[B46] Lin Y. H. Mi F. L. Chen C. T. Chang W. C. Peng S. F. Liang H. F. (2007). Preparation and characterization of nanoparticles shelled with chitosan for oral insulin delivery. *Biomacromolecules* 8 146–152. 10.1021/bm0607776 17206800

[B47] Lin Y.-S. Chen Y.-H. (2017). Biodegradable nanocomplex. U.S. Patent No 20170043007. Washington, DC: U.S. Patent and Trademark Office.

[B48] Martin J. Wilcox M. (2016). New and emerging therapies for *Clostridium difficile* infection. *Curr. Opin. Infect. Dis.* 29 546–554. 10.1097/QCO.0000000000000320 27753689

[B49] Maynard-Smith M. Ahern H. McGlashan J. Nugent P. Ling R. Denton H. (2014). Recombinant antigens based on toxins A and B of *Clostridium difficile* that evoke a potent toxin-neutralising immune response. *Vaccine* 32 700–705. 10.1016/j.vaccine.2013.11.099 24342251 PMC3969267

[B50] Mishra N. Goyal A. K. Tiwari S. Paliwal R. Paliwal S. R. Vaidya B. (2010). Recent advances in mucosal delivery of vaccines: role of mucoadhesive/biodegradable polymeric carriers. *Expert Opin. Ther. Pat.* 20 661–679. 10.1517/13543771003730425 20345332

[B51] Monot M. Eckert C. Lemire A. Hamiot A. Dubois T. Tessier C. (2015). *Clostridium difficile*: new insights into the evolution of the pathogenicity locus. *Sci. Rep.* 5:15023 10.1038/srep15023 PMC4597214 26446480

[B52] Moon H. J. Lee J. S. Talactac M. R. Chowdhury M. Y. Kim J. H. Park M. E. (2012). Mucosal immunization with recombinant influenza hemagglutinin protein and poly gamma-glutamate/chitosan nanoparticles induces protection against highly pathogenic influenza A virus. *Vet. Microbiol.* 160 277–289. 10.1016/j.vetmic.2012.05.035 22763171

[B53] Mulligan M. E. Miller S. D. McFarland L. V. Fung H. C. Kwok R. Y. (1993). Elevated levels of serum immunoglobulins in asymptomatic carriers of *Clostridium difficile*. *Clin. Infect. Dis.* 16(Suppl. 4) S239–S244. 8324125 10.1093/clinids/16.supplement_4.s239

[B54] Pechine S. Collignon A. (2016). Immune responses induced by *Clostridium difficile*. *Anaerobe* 41 68–78. 10.1016/j.anaerobe.2016.04.014 27108093

[B55] Reddy S. T. van der Vlies A. J. Simeoni E. Angeli V. Randolph G. J. O’Neil C. P. (2007). Exploiting lymphatic transport and complement activation in nanoparticle vaccines. *Nat. Biotechnol.* 25 1159–1164. 10.1038/nbt1332 17873867

[B56] Ryan A. Lynch M. Smith S. M. Amu S. Nel H. J. McCoy C. E. (2011). A role for TLR4 in *Clostridium difficile* infection and the recognition of surface layer proteins. *PLoS Pathog.* 7:e1002076 10.1371/journal.ppat.1002076 PMC3128122 21738466

[B57] Sahoo S. K. Panyam J. Prabha S. Labhasetwar V. (2002). Residual polyvinyl alcohol associated with poly (D,L-lactide-co-glycolide) nanoparticles affects their physical properties and cellular uptake. *J. Control. Release* 82 105–114. 12106981 10.1016/s0168-3659(02)00127-x

[B58] Sorg J. A. Sonenshein A. L. (2008). Bile salts and glycine as cogerminants for *Clostridium difficile* spores. *J. Bacteriol.* 190 2505–2512. 10.1128/JB.01765-07 18245298 PMC2293200

[B59] Sougioultzis S. Kyne L. Drudy D. Keates S. Maroo S. Pothoulakis C. (2005). *Clostridium difficile* toxoid vaccine in recurrent *C. difficile*-associated diarrhea. *Gastroenterology* 128 764–770. 15765411 10.1053/j.gastro.2004.11.004

[B60] Spencer J. Leuzzi R. Buckley A. Irvine J. Candlish D. Scarselli M. (2014). Vaccination against *Clostridium difficile* using toxin fragments: observations and analysis in animal models. *Gut Microbes* 5 225–232. 10.4161/gmic.27712 24637800 PMC4063849

[B61] Tian J. H. Fuhrmann S. R. Kluepfel-Stahl S. Carman R. J. Ellingsworth L. Flyer D. C. (2012). A novel fusion protein containing the receptor binding domains of *C. difficile* toxin A and toxin B elicits protective immunity against lethal toxin and spore challenge in preclinical efficacy models. *Vaccine* 30 4249–4258. 10.1016/j.vaccine.2012.04.045 22537987

[B62] Trombetta E. S. Mellman I. (2005). Cell biology of antigen processing in vitro and in vivo. *Annu. Rev. Immunol.* 23 975–1028. 10.1146/annurev.immunol.22.012703.104538 15771591

[B63] Wang H. Sun X. Zhang Y. Li S. Chen K. Shi L. (2012). A chimeric toxin vaccine protects against primary and recurrent *Clostridium difficile* infection. *Infect. Immun.* 80 2678–2688. 10.1128/IAI.00215-12 22615245 PMC3434558

[B64] Wang Y. K. Yan Y. X. Kim H. B. Ju X. Zhao S. Zhang K. (2015). A chimeric protein comprising the glucosyltransferase and cysteine proteinase domains of toxin B and the receptor binding domain of toxin A induces protective immunity against *Clostridium difficile* infection in mice and hamsters. *Hum. Vaccin. Immunother.* 11 2215–2222. 10.1080/21645515.2015.1052352 26036797 PMC4635733

[B65] Warny M. Vaerman J. P. Avesani V. Delmee M. (1994). Human antibody response to *Clostridium difficile* toxin A in relation to clinical course of infection. *Infect. Immun.* 62 384–389. 8300199 10.1128/iai.62.2.384-389.1994 PMC186119

[B66] Xiang S. D. Scholzen A. Minigo G. David C. Apostolopoulos V. Mottram P. L. (2006). Pathogen recognition and development of particulate vaccines: does size matter? *Methods* 40 1–9. 10.1016/j.ymeth.2006.05.016 16997708

[B67] Yue Z. G. Wei W. Lv P. P. Yue H. Wang L. Y. Su Z. G. (2011). Surface charge affects cellular uptake and intracellular trafficking of chitosan-based nanoparticles. *Biomacromolecules* 12 2440–2446. 10.1021/bm101482r 21657799

[B68] Zhao K. Zhang Y. Zhang X. Shi C. Wang X. Wang X. (2014). Chitosan-coated poly(lactic-co-glycolic) acid nanoparticles as an efficient delivery system for Newcastle disease virus DNA vaccine. *Int. J. Nanomed.* 9 4609–4619. 10.2147/IJN.S70633 PMC4207079 25356070

[B69] Zhao S. Ghose-Paul C. Zhang K. Tzipori S. Sun X. (2014). Immune-based treatment and prevention of *Clostridium difficile* infection. *Hum. Vaccin. Immunother.* 10 3522–3530. 10.4161/21645515.2014.980193 25668664 PMC4514135

