# Supplementary material for: Immunization with Recombinant TcdB-Encapsulated Nanocomplex Induces Protection against Clostridium difficile Challenge in a Mouse Model
Source: Front Microbiol. 2017 Jul 25;8:1411. doi: 10.3389/fmicb.2017.01411 (PMC5525027; doi:10.3389/fmicb.2017.01411)

Supplementary Figure 1. Fecal spore count of nanoparticle vaccinated mice following *C. difficile* challenge. *C. difficile* shedding in mouse fecal samples from immunized and control mice following orogastric challenge with 10^6 CFU of *C. difficile* strain R20291. Results denote fecal shedding of *C. difficile* reported as CFU per gram (the geometric mean plus standard error of the mean) from fecal pellets. One fecal pellet was collected 2 days post-challenge from every mouse in each group containing five mice. Statistical comparisons between vehicle and other groups were analyzed by Student's *t*-test.

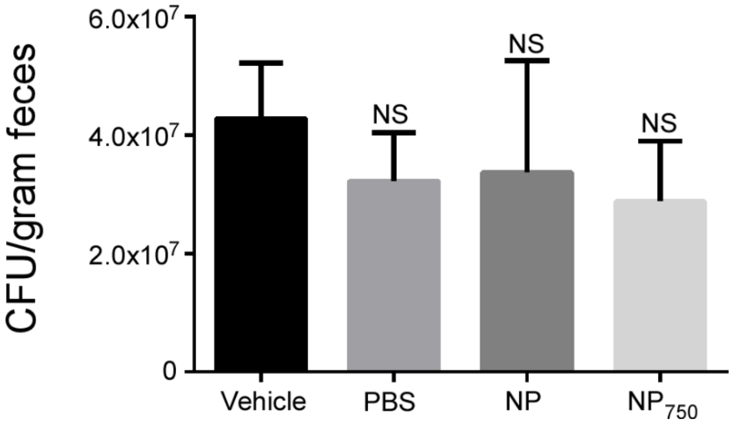

Supplement: Supplementary file 1 [file Image_1.PDF]
